# Food for Thought: Optical Sensor Arrays and Machine Learning for the Food and Beverage Industry

**DOI:** 10.1021/acssensors.4c00252

**Published:** 2024-04-10

**Authors:** William J Peveler

**Affiliations:** † School of Chemistry, Joseph Black Building, University of Glasgow, Glasgow, G128QQ U.K.

**Keywords:** sensing array, cross-reactive, electronic nose, machine learning, food, beverages, smell, taste

## Abstract

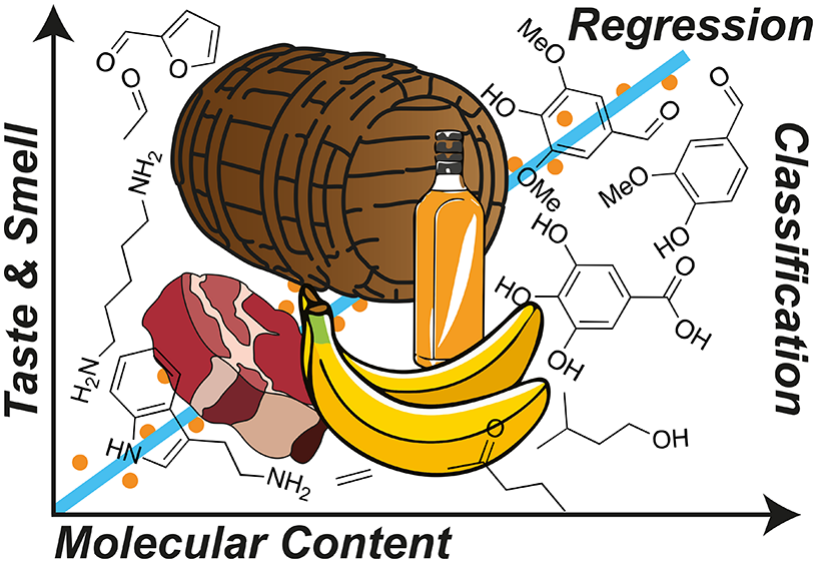

Arrays of cross-reactive sensors, combined with statistical or machine learning analysis of their multivariate outputs, have enabled the holistic analysis of complex samples in biomedicine, environmental science, and consumer products. Comparisons are frequently made to the mammalian nose or tongue and this perspective examines the role of sensing arrays in analyzing food and beverages for quality, veracity, and safety. I focus on optical sensor arrays as low-cost, easy-to-measure tools for use in the field, on the factory floor, or even by the consumer. Novel materials and approaches are highlighted and challenges in the research field are discussed, including sample processing/handling and access to significant sample sets to train and test arrays to tackle real issues in the industry. Finally, I examine whether the comparison of sensing arrays to noses and tongues is helpful in an industry defined by human taste.

The food and beverage industries are a complex, multitrillion-dollar network of raw materials, semifinished, and consumer products that spans hugely differing values from the simplest daily staples up to the priciest Veblen goods. Within this vast industry, sensing and measurement is crucial to monitor materials safety (e.g., to check for spoilage or contamination), to assess quality and integrity (if the material is what it claims to be and if it is from where it claims to be from) and to lead product design (taste, texture, appearance, longevity).

Typically, the need for metrology increases with increasing product value (risk of fraud), increased material/product heterogeneity (trying to “normalize” batches of a product), or where human factors (consumer preference, nutritional value) and legal ramifications (safety requirements, allergens designated or protected status) are considered.^1^ However, sensing in food and drinks can be incredibly complex. Simpler measures of color/optical density, pH, or mechanical properties are routine. But to understand the hugely complex matrix of chemistries contained in a food or beverage ingredient or product that contribute to flavor or spoilage, much more sophisticated methods are required.

Techniques such as mass spectrometry (MS) linked with chromatography (gas, liquid, supercritical fluid driven) or nuclear magnetic resonance spectroscopy (NMR) can be used to directly analyze as many of the chemical species in a sample as possible (“sensomics”), but given the large quantities of complex spectrometric/spectroscopic data produced, the advent of easily applied statistical and machine learning techniques (“chemometrics”) has been crucial to make the most of the data.^2^

By way of example, Uhrín and co-workers have specialized in the direct NMR spectroscopy and mass spectrometry measures of Scotch whisky demonstrating methods for congener (flavor molecule) elucidation,^3,4^ classification against flavor and production method,^5^ and method development in high resolution mass spectrometry to better analyze congeners and better understand product appearance and stability.^6^

Untargeted liquid chromatography/mass spectrometry methods have been used to analyze off-flavors in coffee beans against professional perception scores (here, the Specialty Coffee Association cup scores). Peterson and co-workers were able to use a machine learning model (orthogonal projection to latent structures OPLS regression, *vide infra*) to identify four key compounds (out of hundreds identified) that might negatively impact coffee flavor and quantify these in roasted and unroasted beans as a putative early indicator of quality.^7^

Other direct mass spectrometry methods such as matrix-assisted laser desorption ionization (MALDI) have been demonstrated in fingerprinting the authenticity and origin of products, such as olive oil. Zambonin and co-workers measured phospholipid profiles with MALDI mass spectrometry to analyze the presence of hazelnut oil in adulterated extra virgin olive oil samples,^8^ and Kuo et al. used MALDI to measure triacylglycerol fingerprints in a wide variety of edible oil mixtures to classify and quantify adulterants.^9^

This is just a tiny sample of the work being done in this space, but while such leaps in matching detailed molecular structure and concentration information (molecular fingerprints) with perception or veracity data with are hugely valuable, the barrier to access the required instrumental methods is high. With extensive set up and running costs and the need for highly trained personnel, setting up these methods across production sites or at the point-of-need can be challenging. So, the questions arising are “can simpler, lower-cost methods provide high quality chemical information on food and beverage samples?” and; “can these approaches be applied at “point-of-need”, in goods-in, on the factory floor, or at the point-of-sale, where there may only be limited laboratory access or no laboratory at all?”

## Addressing Sensing Challenges in the Food and Beverage Industry

Two approaches have been taken by researchers to try to address these questions. The first approach is the use of direct (miniaturized/portable) spectroscopy with diagnostic molecular detection potential. Optical approaches often have a lower setup and running costs, and are easier to shrink into a hand-held device. Examples include excitation emission spectroscopy, Raman spectroscopy or Infrared spectroscopy (IR).^10^ In each case, statistical or machine learning-enabled deconvolution of the output spectra can be used to identify certain optically active components within a sample or reference a sample more broadly against a database for classification. The amount of chemical “omic” information is lower than for MS-coupled methods but still high, and the approach is typically very rapid (minutes or seconds). There can still be a need for more expensive excitation sources, cameras or detectors to get the most detailed information, but rapid leaps in miniaturization are reducing cost and making these approaches more popular in the QC laboratory and even amenable to online or *operando* applications (e.g., hyperspectral imaging built into a production line).^11^ The ability to undertake remote or spatially offset (noninvasive) testing can be a benefit, and suites of spectral analysis libraries and toolboxes are becoming available. A detailed discussion of the potential of direct portable optical testing can be found in several recent books and reviews,^12,13^ and here we will limit our discussion to its use as a transducer in the read out of the second common approach, chemical sensing arrays.

**Figure 1:**
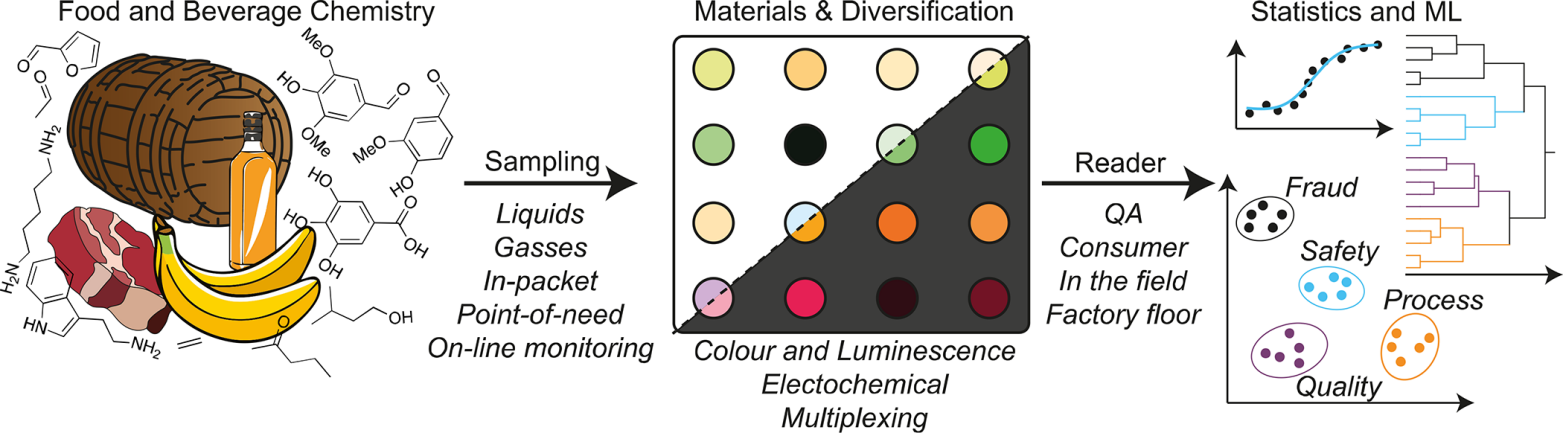
Overview of sensing arrays in food and beverage analysis. Sensing arrays offer distinct advantages in analyzing the sensomic profiles of food and beverages in minimal format that can be included in packaging or used on the factory floor. Many different types of arrays with many different outputs can be tailored to the application in hand and read out by a consumer or an operator monitoring a process. Sensors can be designed for quality assurance (QA), safety and spoilage, or food fraud applications.

This second approach, the focus of this Perspective, is the cross-reactive chemical sensing array (Figure 1). A group or array of different sensors are exposed to a sample, and each sensor reacts with components of the sample to generate a collective response.^14^ Samples are typically complex mixtures of chemical compounds (e.g., a foodstuff or a beverage), and the chemical reactivity can be one or any combination of adsorption to a surface, a redox process, a specific (bio)molecular interaction, a supramolecular interaction, and so on. The key to this approach is that different parts of the array will react with different components within the mixture (cross-reactivity), and measuring each element of the array holistically generates a sensing “fingerprint” for the mixture.

## Cross-Reactive Sensor Array Construction, Materials and Analytical Outputs

The cross-reactive sensing approach has been applied widely across (bio)analytical chemistry, with the individual sensors in the arrays constructed from reactive small molecules,^15^ macrocyclic or polymeric supramolecular systems,^16,17^ nanoscale materials,^18^ or engineered biomacromolecules.^19,20^ To generate a response from the array to the chemistry contained within the sample, a range of binding or bonding interactions can be probed, for example, dispersion forces, hydrogen bonding, charge, or hydrophobic/hydrophilic interactions. Patterns of receptors (e.g., polymers or nanoparticles with a particular repeat or surface unit) or preorganized receptors (e.g., cavitands) can increase specificity for elements in the array to key families of molecules in the sample.^16^ Chemical reactivity (redox chemistry, formation of dynamic or stable dative or covalent bonds) can also be exploited, increasing array response and diversity, although the increased irreversibility of these reactions lend themselves best to a one-time-use sensor array.^21^

The readout of the array can be achieved using a wide range of optoelectronic transduction methods depending on the sensors used, including electrochemical voltammetry, amperometry or impedance; or optical absorbance, luminescence or vibrational spectroscopy.^1^ The output at each array element is combined into a multivariate pattern for the sample, and many different samples can be measured with the same array to generate many different patterns based on their chemical composition and reactivity. The similarities or differences between these patterns can then be interrogated using statistical or machine learning tools, using the tools of chemometrics.^22^

The patterns derived from the sensors are largely analyzed with computationally inexpensive linear transformation methods such as Principal Component Analysis (PCA) to examine which elements in an array contribute to the similarities or differences between samples observed (an unsupervised approach), or its close relation, Linear Discriminant Analysis (LDA), optimized for classification with data labels provided by the user (a supervised approach).^22^ Clustering analyses (e.g., unsupervised hierarchical clustering analysis, HCA) have also been widely applied and have the advantage of defining many levels of structure or similarity in the data beyond simple nearest neighbor analysis. Regression is also increasingly valued, and methods such as partial least-squares (or projection to latent structures) regression (PLS) and orthogonal projections to latent structures (OPLS) are increasingly applied to spectral data outputs.^23,24^ With the rise of increased computer power, and larger, more diverse data sets, there is also a growing move to more capable but perhaps less transparent and more easily overfitted, supervised machine learning methods including support vector machines (SVM), random forests and artificial neural nets (ANNs).^25^

Arguably the earliest examples and certainly the highest TRL (technology readiness level) sensing arrays applied in the food and beverage industry are cross-reactive electrochemical gas sensors as “chemical noses”.^26^ These sensors are largely based around adsorption of volatile species to arrays of chemiresistive materials, with various chemical reactivities (acidic, basic, oxygen rich/poor) and filter layers added on top. The measurement of the resistivity across each array element comprises the sample fingerprint. These arrays originally made use of metal oxide semiconductor materials, operating at high temperatures (several hundred degrees centigrade), but more recently arrays of modified carbonaceous materials such as carbon nanotubes (CNTs) have become popular thanks to successful operation at or close to room temperature (making them easier to build into portable devices).^27^ Such sensors are best applied when there is a good set of volatiles available from the food or beverage (to avoid the need to volatilize the foodstuff or beverage via heating or other means). For example, Swager and co-workers have classified cheeses and liquors, among other foodstuffs/beverages, based on their volatile profiles, using arrays of CNTs decorated with 20 “selector” molecules designed to increase interactions between the CNTs and the sulfur compounds, alcohols, carbonyls, alkanes, and aromatic compounds in the target samples. They analyzed the array outputs with nearest neighbor analysis (kNN) and random forests, with good success for cheese classification, and moderate success for liquors and edible oils.^28^

The other common approach is to create an optode array. These arrays are constructed from different, optically active materials, that respond to the molecules in a sample with an optical wavelength shift (change in color), or change in luminescent intensity (either steady-state or lifetime changes).^21^ Chromophores can be modulated via change of local environment (e.g., displacement from a supramolecular host by an analyte molecule),^16,29^ or chemical reactivity between analytes and the array.^14,25^ Examples include ligation of metalloporphyrins to create a change in absorbance/reflectance;^30^ the reaction or borylated fluorophores with sugars to alter their luminescence;^31^ the change of local polarity and charge around environmentally responsive fluorophores such as coumarins, fluorescent polymers or fluorescent proteins to change their color and intensity;^32^ or the change of the size, shape or local refractive index around a plasmonic nanoparticle to change their plasmonic color.^33−35^ The sensor elements can be immobilized on a solid paper or glass substrate or suspended in solution before addition of the sample (typically in gas or liquid form) to generate the optical response in each array element.

The responses generated can be measured across the array by eye if the changes are clear and obvious enough, but more commonly, simple cameras are used with red-green-blue (RGB) colorimetric analysis, or UV-visible-IR absorbance or luminescence spectroscopy, to measure the output of each array element. The array format lends itself well to high throughput read-out methods, such as wide-field and/or hyperspectral imaging, or well-plate-style, serialized readout formats.^36^ The optical response of multiple luminescent sensor elements can be highly multiplexed in a single location, further reducing the number of array elements that need to be measured and the volume of sample required (potentially down to a whole array in one few-μL well of a 384 well plate).^25,37^ The spectroscopic methods required enable the use of portable spectroscopy via simple illumination sources and basic lenses and gratings for spectroscopic analysis with a CCD or PMT photodetector, or even cellphone cameras^1,38^

The analysis of the optode signals generated by the array can be achieved with the chemometric tools described above, with either point color (RGB)/wavelength changes analyzed with supervised or unsupervised discriminant or clustering methods or a full spectral analysis with partial least-squares methods. The “depth” of data versus the number of different samples and groupings is worth considering when choosing an analysis method, to ensure the method used is suitable for the data acquired (and assumptions of the method are not violated, or overfitting does not occur).^39^

A final consideration when sensor arrays and statistical/machine learning are applied to food and beverages is how the array and analyte are placed in contact with each other. Natural volatiles can be sampled, but if the material is a liquid or solid, it may require dissolution, concentration, or dilution. Liquids can contain the whole sample, including dissolved volatiles, and often have higher concentrations of chemical analytes than those of vapors. Vapor sample concentration is entirely dependent on the relative vapor pressures of the analytes and the sampled headspace. However, vapors do typically present a “matrix free” sample, whereas liquid samples can contain a large background of uninteresting solvent (e.g., water or alcohol) that dilutes and interferes with analyte-sensor interactions. Additionally, liquids or vapors may benefit from some kind of pretreatment to improve the sensing response.^40^ For liquids in particular, (micro)fluidic delivery across a surface enables many spatially separate sensor elements to continuously respond to the sample at once, while minimizing the volume of analyte required, and is becoming a popular approach, alongside microwells.^41^

## Optical Sensor Arrays for Challenges in Food and Beverages

### Classification and Forgery Detection

Many sensor arrays, combined with statistical learning, have been demonstrated for distinguishing between brands, classes, or styles of a particular food or beverage (which may or may not be immediately obvious to the eye, nose, or tongue of the relatively well-educated taster/consumer).

Spirits are a popular target, with many examples of arrays that distinguish between whiskies, baijiu and other fermented and distilled beverages.^42^ The array chemistry should interact with molecules in the beverage under study to detect subtle differences between brands or production styles. For example, whisky is perhaps best distinguished via chemistry arising from its wood aging (tannins, polyphenols, lactones), whereas baijiu can be distinguished via the chemistry arising from the fermentation method and grain used, prior to distillation (esters and organic acids).

**Figure 2:**
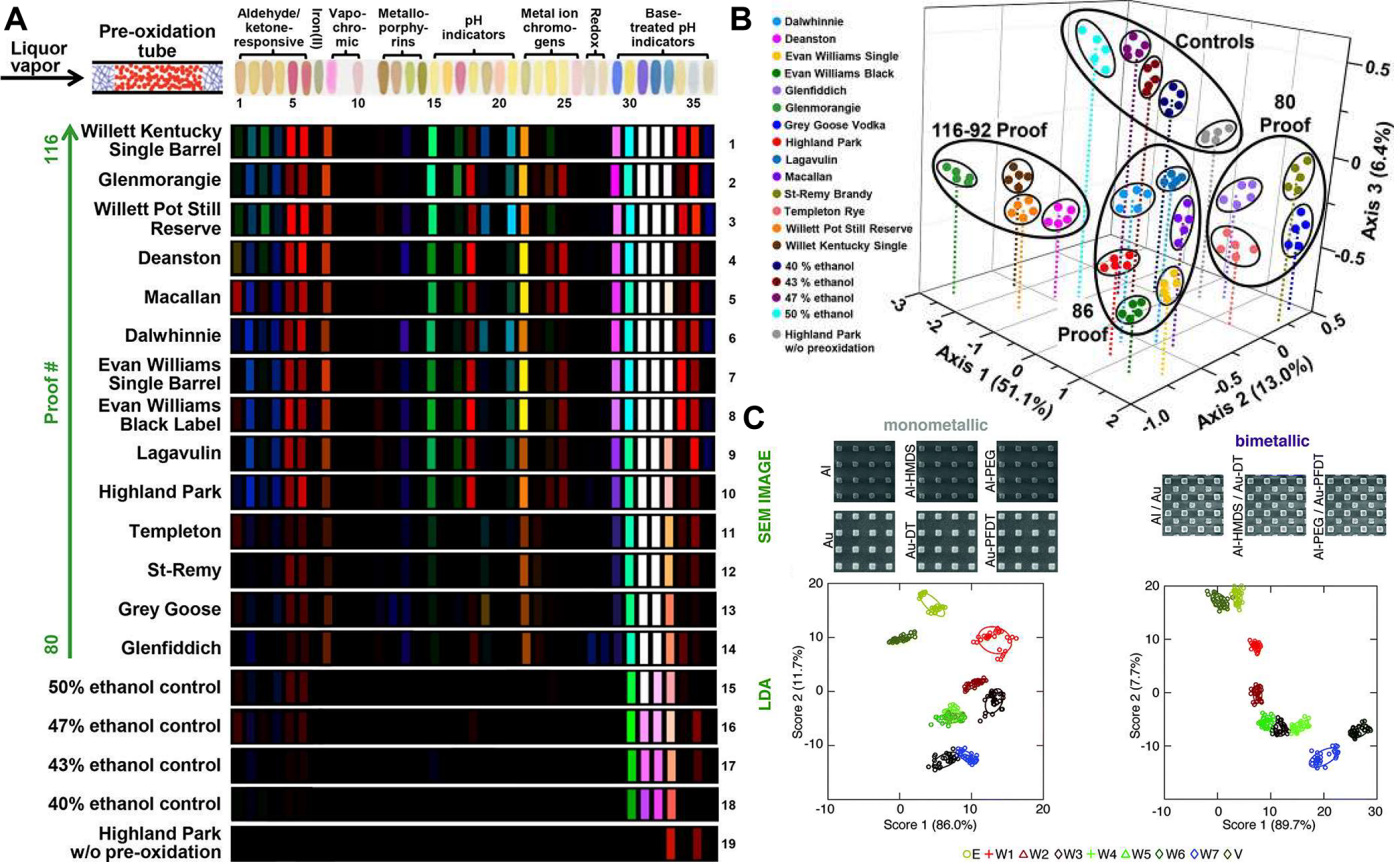
A) RGB colorimetric responses of a 36-element reactive array, measured on a hand-held reader for various spirits after oxidation of their headspace vapors. B) These patterns can be analyzed with PCA to identify factors such as Proof. Adapted with permission from Li & Suslick, ACS Sensors. 2018, 3 (1), 121–127 Copyright 2018 American Chemical Society.^40^ C) Arrays of gold and aluminum nanoparticles on glass can be multiplexed with orthogonal surface chemistries and nonoverlapping optical transmission spectra to create an array capable of discriminating spirits. Adapted from Nanoscale 2019, 11 (32), 15216–15223 under a CC BY 3.0 DEED Unported license.^33^

Li and Suslick demonstrated headspace analysis on various spirits, including whisky, bourbon, and brandy. The volatile alcohols and carbonyls in the headspace were passed over a reactive array of 36 chromophore elements, with a partial preoxidation step (Figure 2A,B). The elements were composed of an array of pH, oxidation/reduction, and base/acid responsive molecular complexes that change color in a differential fashion when exposed to various common functional groups. RGB chromatic shifts (before versus after) across the array were collected on a hand-held reader and analyzed by HCA and SVM to identify 14 spirits from around the world, with the outputs able to distinguish alcoholic strength (proof) as well as sample dilution by as little as 1%.^40^ Suslick has pioneered this style of gas-sensing optical array and previously demonstrated the breadth of the approach, discriminating coffees, beers and many other foodstuffs.^43,44^

Using an alternative approach featuring an array of duplexed plasmonic elements of orthogonally functionalized Au and Al nanoparticles, Clark and co-workers have created a “plasmonic tongue” (Figure 2C). Each plasmonic element generates an LSPR response that is modified by the local dielectric environment, and by coating the plasmonic elements with different chemistries, differential interactions with a sample can be achieved. This red or blue shifts the plasmon response, giving cross-reactive responses from a measurement of the array transmission spectra. The multiplexed nature of the array increased the dimensionality, and discriminating power for age and style, when tested against seven whiskies as well as vodka and 40% ethanol solution.^33^

Baijiu samples have been discriminated using a variety of different arrays based around reactive chromophore generation,^45^ aggregation, growth or etching of silver and gold nanoparticles,^45−47^ luminescent response of lanthanide containing metal organic frameworks,^48^ and the luminescence quenching/enhancement of colloidal quantum dots. In each case, the optical signals were analyzed with PCA, LDA, and HCA and in one case a neural network to distinguish between 12 and 22 brands of baijiu. In several cases the underlying chemistry of the sample impacting the array (e.g., caproic acid, butyric acid, ethyl acetate etc.) was identified and studied in isolation at relevant concentrations.^45^

The spirits described above are often easily distinguished by eye/smell/taste, so in many of these examples, and many more besides,^49^ the need for the sensor array is justified by the need to detect forgery or lower quality products from more expensive or exclusive examples that might be mislabeled.^42^ However, in such cases, if the chemistry sampled by the array is not specific enough to the principal differences between the different classes of product (*i.e*. the chemical differences between brands, makers, styles, or even batches, is greater than the difference between high- or low-quality products), then the sheer scale of the pattern library required to identify all possible products may cause overlaps between “good” and “bad” products.

Furthermore, in many works arrays are trained and tested (clustered or discriminated) against the individual groups or production styles rather than examples of good or bad products, limiting the proof of utility for forgery detection. While “quick and simple” antiforgery applications of sensing arrays in food and beverages are attractive, they are only viable where the need to detect forgery is justifiable and practicable: where there are expensive (Veblen) goods and an extant forgery/black market; high batch to batch consistency of chemistry or clear chemical differences between real and fake goods; and opportunities to sample products on import/export/sale/consumption to actually find the forgeries. Thus, this is not the only area where sensor array research should focus.

### Food and Beverage Quality Assurance

There are many foodstuff and beverage production methods where the chemistry of the sample (and hence the taste and smell) is altered via ingredients choice and product processing, and sensing arrays provide a method for rapidly assessing the quality of input materials that may impact on the downstream process or the success of processes.^2^ This need is greatest where the processes are lengthy, expensive, or otherwise hard to monitor; where they cause clear chemical changes in the sample; and rapid go/no-go decisions might save time, effort, and money. Areas where assurance might be useful (alongside anticounterfeiting, *vide supra*), include rapidly assessing the nutrient content of raw ingredients (vitamins, minerals, antioxidants etc.);^50^ monitoring batch to batch variation in production inside sealed containers (e.g., inside a cask or barrel); or assuring a product has met legal minima, such as minimum aging requirements.^51^

Returning to the example of whisk(e)y, this is a product where the major processing step is long aging in a closed wooden cask for a legally mandated minimum period (3 years in the case of Scotch Whisky, and often much longer) before blending (or vatting) of multiple casks together into a batch for retail. As different casks age at different rates, depending on the wood, cask condition, and storage conditions, they impart different flavor compounds to the spirit. To monitor how the different casks are aging, every cask could be taste tested, but over hundreds of casks in a warehouse or rickhouse, this takes a long time. It is also often impractical or even unsafe to have a local GC or similar tool. Sensing arrays can give a quick holistic impression of a cask based on the reducing chemical content in the aging whisky that stems from the wood contact, including organic acids, polyphenols, furfurals etc. We recently demonstrated a multidimensional sensor that consists of reacting gold or silver salts with different whiskies to create plasmonic nanoparticles as fingerprints for the whisky based on the color of the nanoparticles formed and the rate of their formation, collectively analyzed by HCA. Analysis of a semifinished product, a single cask sampled over time, allowed for matching of the developing chemistry with the sensor array output and development of the sensor as a measure of cask age.^52^

The quantity of antioxidant compounds, particularly flavonoids, tannins, and other phenolic substances, are also important in assessing the quality green and black teas against grading scales, as well as their putative health benefits. For example Huo et al. used a version of Suslick’s color changing arrays to grade and identify geographical origin of nine green teas.^53^ Similarly, the high antioxidant content of a luxury tea (Tieguanyin) was leveraged in an array by Yang et al. to measure different polyphenols in the tea with a peroxidase mimicking metal organic particles and a TMB color changing output, and discriminate genuine from adulterated samples.^54^

**Figure 3:**
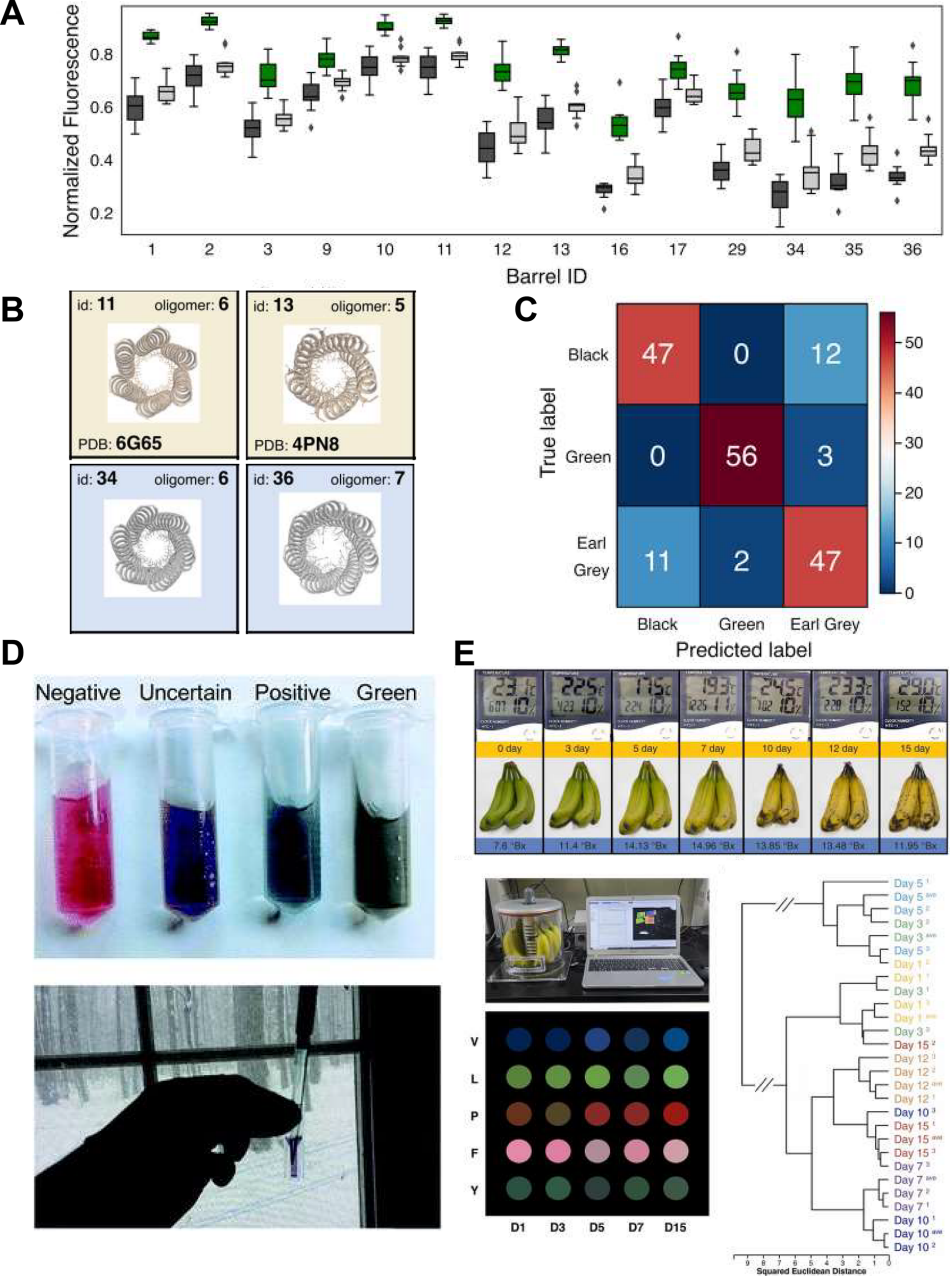
A) Grouped responses to three classes of tea (30 examples) by 14 different proteinaceous barrels (B) with a displaced indicator dye. C) SVM analysis of the fluorescence changes across the array could be used to successfully identify the teas. Adapted Nat Commun 2023, 14 (1), 383 under a CC BY 4.0 license.^19^ D) Large chromatic changes across RGB space by gold nanoparticles in response to off flavors in maple sap and syrup samples enable an array of responses from a single sensor element. The simplicity of the test enables this to be carried out at the point-of-need in rural Canada. Reproduced from Anal. Methods 2020, 12 (19), 2460–2468 with permission from the Royal Society of Chemistry.^57^ E) Structural color created by arrays of functionalized bacteriophages is used to monitor the volatiles created by ripening bananas over 15 days. RGB extraction and HCA analysis can be used to follow the process. Adapted from Kim et al. Sensors and Actuators B 2022, 362, 131763 copyright Elsevier (2022).^58^

Many other examples of sensing teas exist using a versatile set of sensing array construction methods. Ni et al. recently leveraged the reactivity of boronates with catechols to create an indicator displacement assay consisting of combinations of two indicator fluorophores and three multidentate binder/quenchers for various plant derived polyphenols in tea. Various statistical or ML techniques were applied to the data with LDA found to be the most successful in discriminating the 16 teas under test.^55^ Bunz and co-workers used a library of conjugated polymers, some quenched by macrocyclic and cucurbit[8]uril to measure amino acids or xanthines in teas. Molecules such as caffeine and theobromine interacted with the polymers and macrocycle to trigger a differential turn on fluorescent response that could separate the 22 different teas.^56^ Finally, Woolfson and co-workers utilized an array of 14 different coiled coil peptide barrels and an environmentally responsive dye (1,6-diphenyl-1,3,5-hexatriene) to create differential fluorescent responses for 30 teas in three classes (earl gray vs black tea vs green tea) via a support vector classifier (Figure 3A-C).^19^ While these later examples focus mostly on discriminating green from black tea, oolong tea, or other obviously different teas, they do highlight how versatile chemical approaches, particularly using host–guest or dynamic covalent interactions can be combined and applied to create sensing arrays for the same target chemistries.

Maple syrup is an expensive product thanks to the remoteness of the raw materials (in the forests of North America) and the long processing (boiling) step to concentrate tree sap into the final sugary syrup. Sap quality is hugely important because any contaminants or “off-flavors” in the raw sap are unavoidably concentrated into the final syrup, lowering the quality or spoiling the batch. Compounding the difficulties, sap is harvested and processed in large volumes far from laboratories, so a point-of-need testing solution is useful for assessing sap quality before batching and boiling. Masson and co-workers used a cross-reactive gold-nanoparticle aggregation assay to assess the off-note content of sap and finished syrup. Crucially the simple assay has a multicolor read out (Figure 3D, so could be considered a single element array) and can be performed at the point of need in the sugar shacks in rural Quebec. The sample amino acid content was identified as the key consideration for syrup quality and could be measured by the red shifting of gold nanoparticles mixed with sap, using an end-point colorimetric index – COLORI, combined with a mixed effects statistical model to predict likely syrup quality, based on assay data from over 29,000 sap and syrup samples.^57,59^

A final example of quality assurance where arrays have been applied is in monitoring fruit ripening. Many volatiles are released during fruit ripening (including ethylene, 2-pentanone, and 3-methyl-1-butanol) and need to be monitored in transit to avoid spoilage. Oh and colleagues have pioneered colorimetric sensing arrays created from bacterial phages bearing distinct surface peptides, that self-assemble into microscale architectures and display structural color.^20^ The interaction between different phage-based materials and various gases causes material swelling and changes the observed iridescence.^60^ Five differently functionalized phage materials were exposed to fruit ripening gases and ripening bananas, with their color changes monitored by camera and processed to RGB shifts, to successfully follow the ripening process via HCA analysis (Figure 3E).^58^

### Food and Beverage Safety

**Figure 4:**
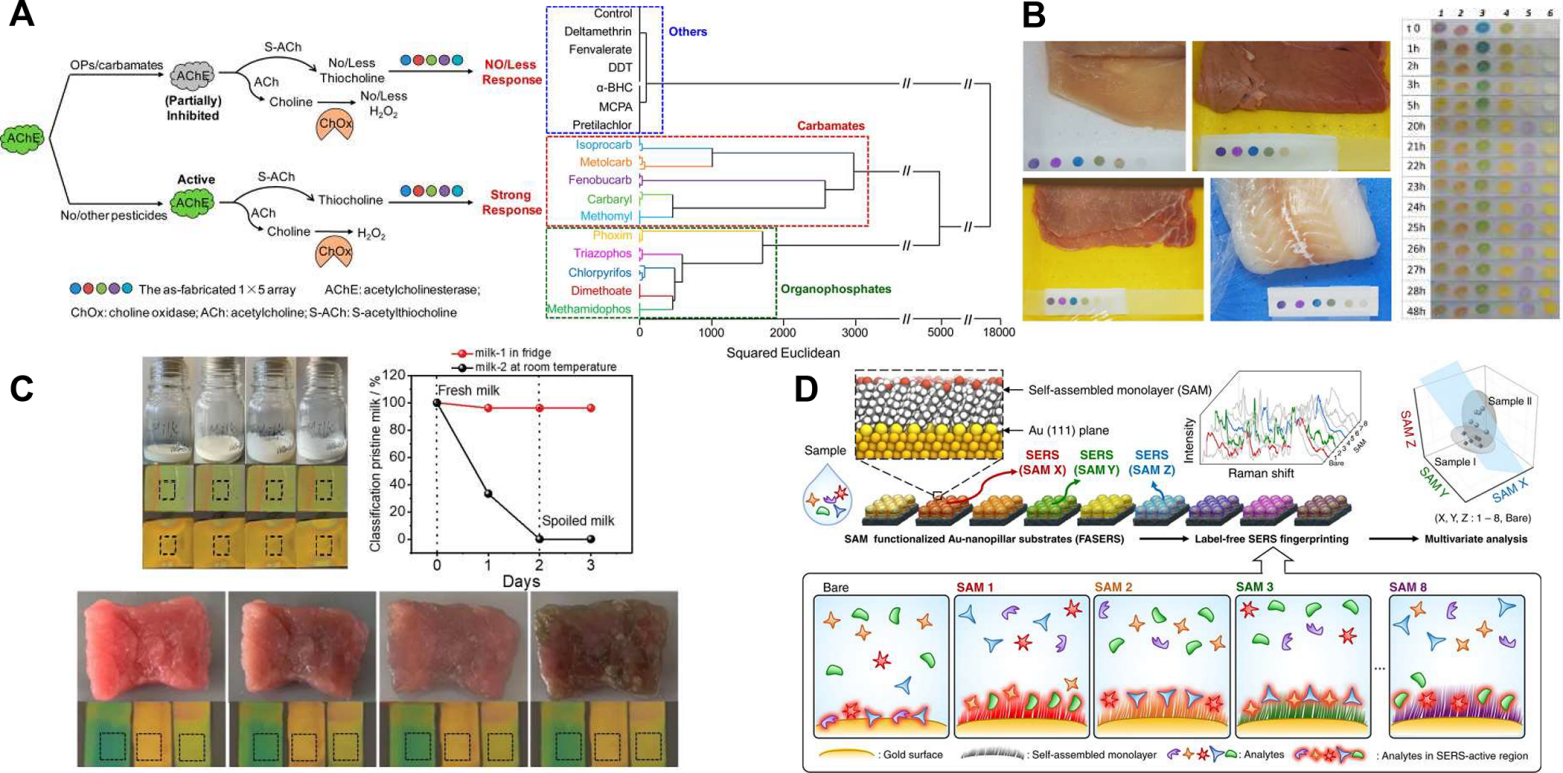
A) Pesticides can be detected and classified by preprocessing samples with a sensitive acetylcholine esterase (AChE) before exposing the products to an array of five compounds that react calorimetrically to any H_2_O_2_ or thiocholine produced by the enzyme. Differential actions of the enzyme and the array are analyzed by HCA to classify different pesticides in apple juice and tea. Adapted with permission from Anal. Chem. 2015, 87 (10), 5395–5400, Copyright 2015 American Chemical Society.^62^ B) Paper strips containing an array of indicator dyes within sealed meat and fish packaging react to volatile compounds to signal spoilage. Analysis of the RGB images by PCA allowed for classification into “safe”, “warning”, and “hazard” groups. Adapted from Foods 2020, 9 (5), 684 under a CC BY license.^66^ C) MOF-based colorimetric sensors for spoilage also react to emitted volatile compounds, and kNN analysis of image color can be used to monitor freshness of milk and meat. Adapted from Adv. Mater. Interfaces 2023, 10 (28), 202300329 under a CC BY 4.0 DEED license.^67^ D) An alternative transduction approach to measuring array-sample interactions is to use surface enhanced Raman spectroscopy on an array of surface modified gold nanopillars. The surface modification differentially alters the interaction between surface and target, and therefore what Raman signals are observed before analysis with PCA and LDA. Adapted from Nat Commun 2020, 11 (1), 207 under a CC BY 4.0 license.^68^

A final exemplar area where sensing arrays can be usefully applied is food and beverage consumer safety. Arrays have been used to detect toxic contaminants such as pesticide residues or heavy metals (Figure 4A),^61,62^ illegally introduced dopants such as melamine in infant milk,^63^ or food-borne pathogens.^64^

Bacterial growth and spoilage of food items such as meat, fish, and dairy poses a high risk to consumers. Markers of spoilage can include biogenic amines such as spermine, organic acids, or thiols. To detect biogenic amines, arrays comprising metal complexes that change their UV-visible spectra have been shown to be effective. Singh et al. built a liquid array and a portable microplate reader that could detect spermine and tryptamine contamination in meat and cheese, although sample preparation was laborious.^65^ In a similar fashion Hormozi-Nezhad and co-workers built a liquid array based on eight surface modified Ag and Au NPs that aggregated and changed their plasmonic color in the presence of biogenic amines in extracted meat samples and would be similarly readable with a portable spectrometer.^34^

Unpowered, solid-state optical arrays that can be read with a camera or by eye offer a huge advantage. They can be incorporated within packaging as externally readable sensors to indicate if the contents have spoiled. Arrays of reactive color-changing ink spots responsive to pH (volatile organic acids and amines) and thiols have been exploited by Magnaghi et al. for defining “safe” “warning” or “hazard” categorizations to meat and fish, that had been left at room temperature, from within the packet (Figure 4B). RGB analysis on smartphone camera images coupled with PCA could warn of spoilage, although these categorizations were not externally defined by other gold standard testing or microbiology.^66^ Heinke and co-workers developed an array of thin metal organic framework (MOF) films that formed Fabry-Pérot cavities when oversputtered with metal (Figure 4C). The porous cavity filling between metal layers means gases entering the MOF material change the observed color of the cavity, allowing for colorimetric gas sensing based on the gases present and their interaction with the varied MOF material. Read out was possible with a smartphone camera, RGB analysis and kNN clustering, and the devices were used to measure the spoilage of milk and meat samples inside containers.^67^

## The Future of Sensor Arrays for Food and Beverages?

In the selection of examples above, I have tried to illustrate the possibilities that chemical sensing arrays, combined with statistical or machine learning can offer sensing in the food and beverage industry, tackling challenges in quality assurance, production, and safety. New materials and approaches to sensing in liquids and gases offer a wealth of cross-reactive or targeted arrays, and a problem-driven approach is key to making useful progress for the industry.

Arrays combined with hand-held/portable electronics and optics are far more suited to point of need testing than the gold-standard omic techniques such as LC, GC, MS and NMR. Although arrays cannot deliver the same untargeted molecular precision as these methods, they can be easily tuned for the samples if the underlying chemical content is taken into consideration when designing and constructing the sensing elements. A move to embracing optical arrays allows for simple analysis using hand-held devices, including smartphones, and the computational power required to collect and analyze array data against a pretrained model or a library of preclassified samples is well within the capabilities of such devices for more simple linear methods such as LDA. Even if more computationally expensive image/video analysis is required or the data load is very high (many samples or time points), calculations can be performed via remote data services.

A current challenge is that many arrays, from the original “e-noses” to the latest plasmonic sensor arrays, rely on a differential adsorption or an increase in local molecular mass at each array element to generate the signals. This can lead to relatively low “orthogonality” in the data generated, limiting the discriminatory power of the array as the number of different samples increases and the similarity between them increases. Chemically reactive arrays that have diverse responses to different chemical moieties are a counter example, but are inherently “one-time” use, which can be a limitation in certain circumstances. Fluorophores that respond in color and intensity to different charge, solvation, and bonding environments can also increase orthogonality, but weaker binding interactions require higher concentrations of target to generate an effect.

New materials being used to construct sensing arrays allow for new modes of operation that may add orthogonality to the array outputs. Steady state luminescence has been thoroughly exploited, but lower cost integrated single photon avalanche detector (SPAD) arrays have the potential to make luminescence lifetime imaging more easily available, multiplying the outputs of suitably designed luminescent array that varies in intensity, emission color, and lifetime(s). Established plasmonic materials (e.g., gold nanoparticles) and newer Raman-active 2D materials (e.g., MXenes) open up the potential for surface enhanced Raman arrays where surface chemistry interacts with molecular targets to not only give a Raman spectrum of the target but alter the enhanced spectrum of the surface coating, giving rise to a highly multidimensional output from the array with potential for “fingerprinting” as well as a degree of direct detection via enhancements in key Raman regions.^68^

Array stability, whether the shelf life of a one-time use array or long-term regenerability of a reusable array, is also a challenge. Library collection and statistical training is only valid if the array and its outputs are reproducible over time, and so simple construction and built-in standardization (either unchanging elements, measurement of standards, or pre/post exposure measurements) are useful to ensure usable data is collected. This is particularly relevant in the food and beverage industries, where in-line testing is attractive for many processes, so arrays will have to remain useable after extended periods in potentially harsh environments without degrading or fouling. Much can be learned from the application of arrays in medical diagnostics,^69^ however the number of assays required is greater and budgets are typically tighter in food production, so cost and reusability is a major consideration.

To better train arrays for current problems or challenges in the industry, access to relevant and ideally large and varied sample sets is key. This is particularly true for raw ingredients or semifinished products, or spoiled or otherwise contaminated products that would be hard to collect or mimic/spike without stakeholder input. Such samples will enable researchers to go beyond the simple classification of finished consumer products bought in the local store, and begin to tackle more complex challenges, as exemplified well by Masson’s work with the maple syrup industry in Quebec.^59^

Given the analogy between chemical sensor arrays with machine learning, and the mammalian olfaction system perhaps the most exciting and challenging application in food and beverages would be to generate sensors that can truly mimic human smell and taste.^70^ Human noses do not operate like a GC – we simply cannot distinguish that many different but similar compounds, and many of the compounds detected are not necessarily the compounds a human would use to “taste” or distinguish a food product or beverage. By relying on cross-reactivity, identifying key families or members of families of compounds, and linking these to common descriptors, we can holistically sample and distinguish complex mixtures just as a sensing array can. So can we (and should we) try to align the natural and the artificial? Recent work by Fan and co-workers demonstrates a massively increased number of cross-reactive elements that can be electrochemically surveyed on a chip from tens to hundreds or even thousands. Combining this this with a neural network and computer vision they created their version of a “robot dog”, capable of sniffing out different foodstuffs and detecting aging fruit, taking this biomimetic approach to a new extreme.^71^

A sensing array that is well aligned to human tastes and preferences could be invaluable in taste testing new products, aligning the taste of products with the particular taste of a distinct population, and measuring the consistency of the flavor of a batch produced product when then raw materials are subject to change. Many of these measures are currently achieved by human tasting panels or skilled individuals (master blenders at a distillery, for example), and there is a degree of reticence in many parts of the industry that these could or should ever be replaced. And perhaps they cannot; after all, perception of food and drink is so subjective there is always the need for the human element. However, a versatile technology that is well aligned to taste and smell, and can be tailored to the product under test, can make taster’s lives easier. It could enable optimization and parallelization, particularly when working with very strong flavors, semifinished products (that do not yet taste “good”), or when working with potential toxins where humans cannot operate. It is in this space that I propose sensing arrays and machine learning techniques will really impact industry in the future.
